# Development and validation of a nomogram for predicting in-hospital mortality in ICU patients with infective endocarditis

**DOI:** 10.1186/s12911-024-02482-7

**Published:** 2024-03-21

**Authors:** Dongyang Che, Jinlin Hu, Jialiang Zhu, Jun Lyu, Xiaoshen Zhang

**Affiliations:** 1 Department of Cardiovascular Surgery, The First Affiliated Hospital of Jinan University, 510630 Guangzhou, Guangdong Province People’s Republic of China; 2 Department of Cardiovascular Surgery, The Second Affiliated Hospital of Guangzhou, Guangdong Provincial Hospital of Chinese Medicine, University of Chinese Medicine, 510630 Guangzhou, Guangdong Province People’s Republic of China; 3 The First Affiliated Hospital of Jinan University, 510630 Guangzhou, Guangdong Province People’s Republic of China; 4 Department of Clinical Research, The First Affiliated Hospital of Jinan University, 510630 Guangzhou, Guangdong Province People’s Republic of China

**Keywords:** Infective endocarditis, Nomogram, Intensive care unit, Predictive model, Mortality

## Abstract

**Background:**

Infective endocarditis (IE) is a disease with high in-hospital mortality. The objective of the present investigation was to develop and validate a nomogram that precisely anticipates in-hospital mortality in ICU individuals diagnosed with infective endocarditis.

**Methods:**

Retrospectively collected clinical data of patients with IE admitted to the ICU in the MIMIC IV database were analyzed using the Least Absolute Shrinkage and Selection Operator (LASSO) regression to identify potential hazards. A logistic regression model incorporating multiple factors was established, and a dynamic nomogram was generated to facilitate predictions. To assess the classification performance of the model, an ROC curve was generated, and the AUC value was computed as an indicator of its diagnostic accuracy. The model was subjected to calibration curve analysis and the Hosmer–Lemeshow (HL) test to assess its goodness of fit. To evaluate the clinical relevance of the model, decision-curve analysis (DCA) was conducted.

**Results:**

The research involved a total of 676 patients, who were divided into two cohorts: a training cohort comprising 473 patients and a validation cohort comprising 203 patients. The allocation ratio between the two cohorts was 7:3. Based on the independent predictors identified through LASSO regression, the final selection for constructing the prediction model included five variables: lactate, bicarbonate, white blood cell count (WBC), platelet count, and prothrombin time (PT). The nomogram model demonstrated a robust diagnostic ability in both the cohorts used for training and validation. This is supported by the respective area under the curve (AUC) values of 0.843 and 0.891. The results of the calibration curves and HL tests exhibited acceptable conformity between observed and predicted outcomes. According to the DCA analysis, the nomogram model demonstrated a notable overall clinical advantage compared to the APSIII and SAPSII scoring systems.

**Conclusions:**

The nomogram developed during the study proved to be highly accurate in forecasting the mortality of patients with IE during hospitalization in the ICU. As a result, it may be useful for clinicians in decision-making and treatment.

**Supplementary Information:**

The online version contains supplementary material available at 10.1186/s12911-024-02482-7.

## Background

Infective endocarditis (IE) is a disease in which pathogenic microorganisms such as bacteria or fungi attach to the endocardial tissue of the heart, causing inflammation and damage to the endocardium or valve tissue. The incidence of IE is 3–10 per 100,000 people, with an increasing trend in some places [1, 2]. In spite of various diagnostic tools and therapeutic measures such as antibiotics and surgery being developed, the in-hospital mortality rate due to IE remains at approximately 20% [3–5]. Individual patient characteristics, along with cardiac and non-cardiac comorbidities, the infecting microbial species and the echocardiographic disease status are the primary factors contributing to the poor prognosis of IE [6]. Early diagnosis is now recognized as critical to improving patient outcomes and reducing mortality associated with IE [7].

Due to the complex and unpredictable clinical characteristics and disease progression of IE, timely and accurate diagnosis is imperative to ensure that patients receive appropriate treatment during critical stages of the disease. However, there is currently limited evidence regarding the effective intensive care management of IE, including specific clinical features that are indicative of a poor prognosis, as well as predictive tools that are effective in identifying high-risk patients [8].

A nomogram is a common statistical tool used in medicine to predict the probability or risk of a certain outcome based on multiple patient factors. This tool is especially useful in clinical predictive modeling, as it visualizes the impact of each predictor on the outcome event. Clinicians can use this tool to make prognostic predictions more accurately [9, 10]. The objective of this study was to construct a nomogram utilizing data sourced from the public database MIMIC-IV, subsequently assessing its prognostic value in predicting the risk of in-hospital mortality among patients with IE who were admitted to the ICU.

## Data and method

### Data source

The information utilized in this research endeavor was sourced from MIMIC-IV 2.0, a publicly available archival system for extensive care medicinal data, which was established by the Massachusetts Institute of Technology (Cambridge, MA). The database containing hospitalization data for patients treated at Beth Israel Deaconess Medical Center (Boston, MA) between 2008 and 2019 exists as a comprehensive collection of patient information, and includes vital signs, laboratory test results, organ failure score, disease severity score, comorbidities, diagnosis, treatment, length of hospital stay, survival data and demographic information. The database contains patient information that has been de-identified, and informed consent from patients is not necessary. The authors of this study have completed the requisite training and certification to access the database.

### Study population

**Fig. 1:**
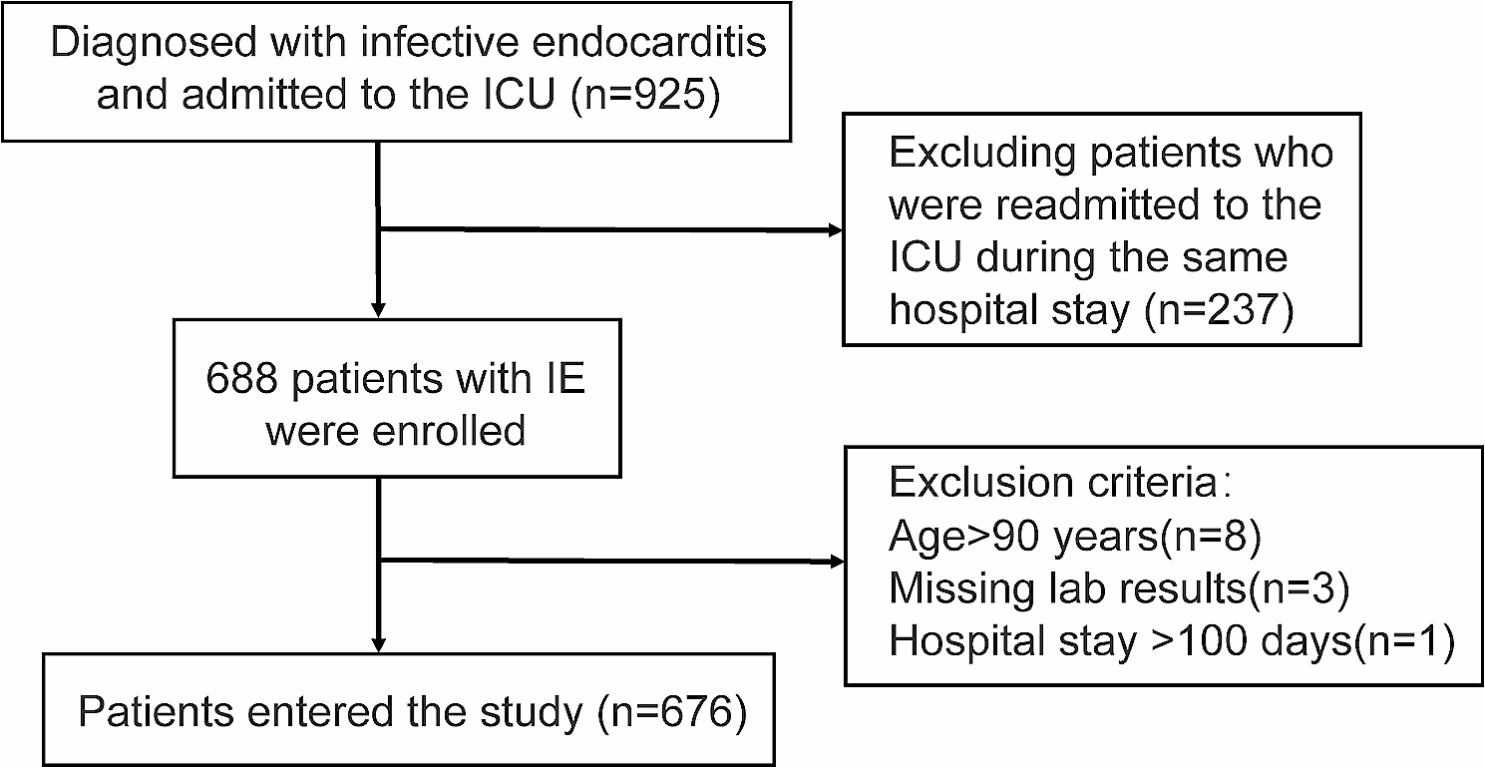
Flow diagram of patient selection (MIMIC IV, Medical Information Mart for Intensive Care IV)

From the MIMIC-IV database, a cohort of 925 patients diagnosed with IE and admitted to the Intensive Care Unit (ICU) was identified. Among them, 237 cases with repeated ICU admissions in a single hospitalization were excluded from the analysis. Patients over 90 years of age, those without basic laboratory test results, and individuals with hospitalization times exceeding 100 days were also excluded from the study. The final analysis consisted of 676 patients deemed eligible according to the pre-determined criteria (Fig. 1).

### Clinical variables

The raw data of the 676 selected patients was extracted using Structured Query Language (SQL) and PostgreSQL tools (version 9.6) through Navicat Premium software, based on their unique HADM_ID and ICUSTAY_ID. The extracted raw data comprised demographic characteristics (age, gender, race and weight), vital signs (heart rate, blood pressure, and temperature), blood gas analysis (lactate, SpO2, SpCO2 and anions gap), laboratory tests (e.g., white blood cells [WBC] and red blood cells [RBC] counts, hemoglobin, prothrombin time [PT], calcium, bicarbonate, glucose, lymphocytes), comorbidities (e.g., atrial fibrillation, myocardial infarction, chronic lung disease, heart valve disease, cerebrovascular disease), score systems (Acute Physiology Score III [APSIII], Simplified Acute Physiology Score II [SAPSII] and Sequential Organ Failure Score [SOFA]), clinical risk factors associated with IE (continuous renal replacement therapy [CRRT], blood culture results, the presence of embolism, history of prior cardiac surgery).

### Statistical analysis

Indexes with a missing degree above 20% were excluded from this study, and remaining missing data were imputed using R packages “lattice (0.21-8)” and “mice (3.16.0)” for multiple imputation. A random allocation of participants into the training and validation cohorts was accomplished in a ratio of 7:3. More specifically, 70% of the study’s subjects were assigned to the training cohort, and the remaining 30% were allocated to the validation cohort to test the data. The median and interquartile range were used to express continuous variables, and the Wilcoxon rank-sum test was employed to compare two groups. Proportions were utilized to represent categorical variables, and the comparison between groups was analyzed by either the Chi-square test or Fisher’s exact test, depending on the context of the comparison. The methodology employed adheres to academic standards in statistical analysis. The variables in the training cohort were screened for mortality predictors using the Least Absolute Shrinkage and Selection Operator (LASSO) approach. The variable set for the logistic regression models was determined through the selection of lambda.1se using cross-validation [11, 12]. Subsequently, utilizing the selected set of variables, we constructed multiple logistic regression models, and nomograms were created using the ‘regplot (1.1)’ R package. The nomogram was validated using data from the validation cohort. The evaluation of the model’s performance was conducted by computing the area under the receiver operating characteristic (ROC) curve, commonly referred to as AUC. Additionally, we compared the AUC of our model with the APSIII and SAPSII scoring systems to evaluate its efficacy.

The utilization of the calibration curve aimed to evaluate discrepancies between the expected outcomes of the model and the factual observed measurements. The assessment was conducted to ensure the accuracy of the model’s predictions in an empirical manner. The Hosmer-Lemeshow goodness-of-fit test (HL test) was employed to determine whether the model’s estimation of the observed probability was consistent with the actual probability. The Integrated Discrimination Improvement (IDI) method was utilized to evaluate the overall improvement in the accuracy of predictions. Furthermore, to establish the clinical validity of the model, a Decision Curve Analysis (DCA) approach was employed. The statistical analyses were performed utilizing the R language (4.3.0), while the level of significance was established at a *p*-value of less than 0.05.

## Results

### Patient characteristics

The eligibility criteria were met by a cohort of 676 patients diagnosed with IE, who were subsequently divided into two cohorts, namely the training cohort (*n* = 473) and the validation cohort (*n* = 203), through a random sampling process. Table 1 provides a comprehensive overview of the demographic and clinical features of the respective cohorts. In the training cohort, the median age was 58 years (43, 68), and the sample consisted of 310 (65.54%) male and 163 (34.46%) female patients. The validation cohort had a median age of 57 years (40, 66), with 132 (65.02%) male and 71 (34.98%) female participants. The majority of patients in both groups were white (> 67%). In the training cohort, the median weight of the patients was 77.3 kg (67.5,92.0). While in the validation cohort, the patients had a median weight of 77.9 kg (66.2, 94.2). Apart from the prevalence of liver disease (*p* = 0.044) (Supplementary materials), baseline clinicopathological data did not significantly differ between the cohorts (*p* = 0.077-1).

**Table 1:** Baseline characteristics of the patients

Variables	Training Cohort	Validation Cohort		*p*-value
*n*	473	203		
**Demographics**				
Age	58.0 [43.0,68.0]	57.0 [40.0,66.5]		0.391
Sex				0.968
Male	310 (65.5%)	132 (65.0%)		
Female	163 (34.5%)	71 (35.0%)		
Race				0.701
White	333 (70.4%)	137 (67.5%)		
Black	41 (8.67%)	21 (10.3%)		
Others	99 (20.9%)	45 (22.2%)		
Weight (kg)	77.3 [67.5,92.0]	77.9 [66.2,94.2]		0.709
**Vital signs**				
HR (times/min)	93.7 [83.9,106]	96.1 [86.2,106]		0.462
SBP (mmHg)	106 [99.8,115]	105 [99.3,113]		0.491
DBP (mmHg)	56.8 [50.9,62.8]	56.5 [50.6,62.6]		0.711
MBP (mmHg)	70.9 [65.7,77.1]	70.3 [64.1,76.1]		0.248
Temperature (°C)	37.1 [36.8,37.5]	37.1 [36.8,37.5]		0.757
**Blood gas**				
Lactate (mmol/L)	2.50 [1.70,3.90]	2.40 [1.50,3.80]		0.166
Spo2 (mmHg)	251 [143,381]	230 [121,372]		0.199
Spco2 (mmHg)	48.0 [41.0,55.0]	48.0 [40.5,56.0]		0.649
AG (mEq/L)	10.0 [8.00,11.0]	10.0 [8.50,12.0]		0.237
**Laboratory test**				
WBC (k/uL)	6.70 [5.00,8.80]	7.00 [4.95,9.20]		0.350
RBC (m/uL)	2.58 [2.31,2.94]	2.60 [2.30,3.08]		0.381
Hemoglobin (g/dL)	7.40 [6.60,8.50]	7.50 [6.60,8.70]		0.519
MCH (IU/mL)	28.3 [26.6,29.8]	28.2 [26.8,29.4]		0.515
Bicarbonate (mEq/L)	20.0 [17.0,23.0]	20.0 [17.0,23.0]		0.529
Chloride (mEq/L)	108 [104,112]	107 [103,111]		0.259
Calcium Total (EU/dL)	7.70 [7.20,8.10]	7.90 [7.30,8.20]		0.077
Potassium (mg/dL)	3.40 [3.10,3.70]	3.40 [3.10,3.70]		0.616
Sodium (mg/dL)	142 [139,146]	142 [139,146]		0.698
Magnesium (mg/dL)	1.70 [1.60,1.90]	1.70 [1.60,1.90]		0.272
Platelet (k/uL)	316 [212,460]	330 [215,454]		0.443
PT(s)	13.1 [12.0,14.4]	12.9 [11.8,14.5]		0.407
PTT(s)	27.5 [25.3,30.5]	27.1 [25.0,30.4]		0.423
ALT (IU/L)	37.0 [21.0,70.0]	33.0 [18.0,73.0]		0.290
LD (IU/L)	341 [262,536]	350 [252,536]		0.939
BUN (mg/dL)	36.0 [22.0,66.0]	35.0 [21.0,66.0]		0.705
Creatinine (g/dL)	1.50 [1.10,3.10]	1.50 [1.10,3.00]		0.988
Glucose(mg/dL)	168 [137,240]	175 [136,234]		0.948
Lymphocytes(%)	13.9 [9.70,21.1]	13.0 [8.75,19.0]		0.142
Monocytes(%)	6.30 [4.40,9.40]	6.00 [4.20,9.00]		0.290
Neutrophils(%)	85.0 [79.7,89.7]	85.0 [79.8,89.0]		0.434
**Scoring systems**				
APSIII	50.0 [36.0,68.0]	48.0 [35.0,65.5]		0.192
SAPSII	34.0 [24.0,44.0]	32.0 [23.5,41.5]		0.084
SOFA	6.00 [3.00,10.0]	6.00 [3.00,9.00]		0.170

HR heart rate; SBP systolic blood pressure; DBP diastolic blood pressure; MBP mean blood pressure; AG anion gap; WBC white blood cell; RBC red blood cell; MCH mean corpuscular hemoglobin; PT prothrombin time; PTT partial thromboplastin time; ALT alanine transaminase; LD lactic dehydrogenase; APSIII Acute Physiology Score III; SAPSII Simplified Acute Physiology Score; SOFA Sequential Organ Failure Assessment

### Variable analysis and selection

**Fig. 2:**
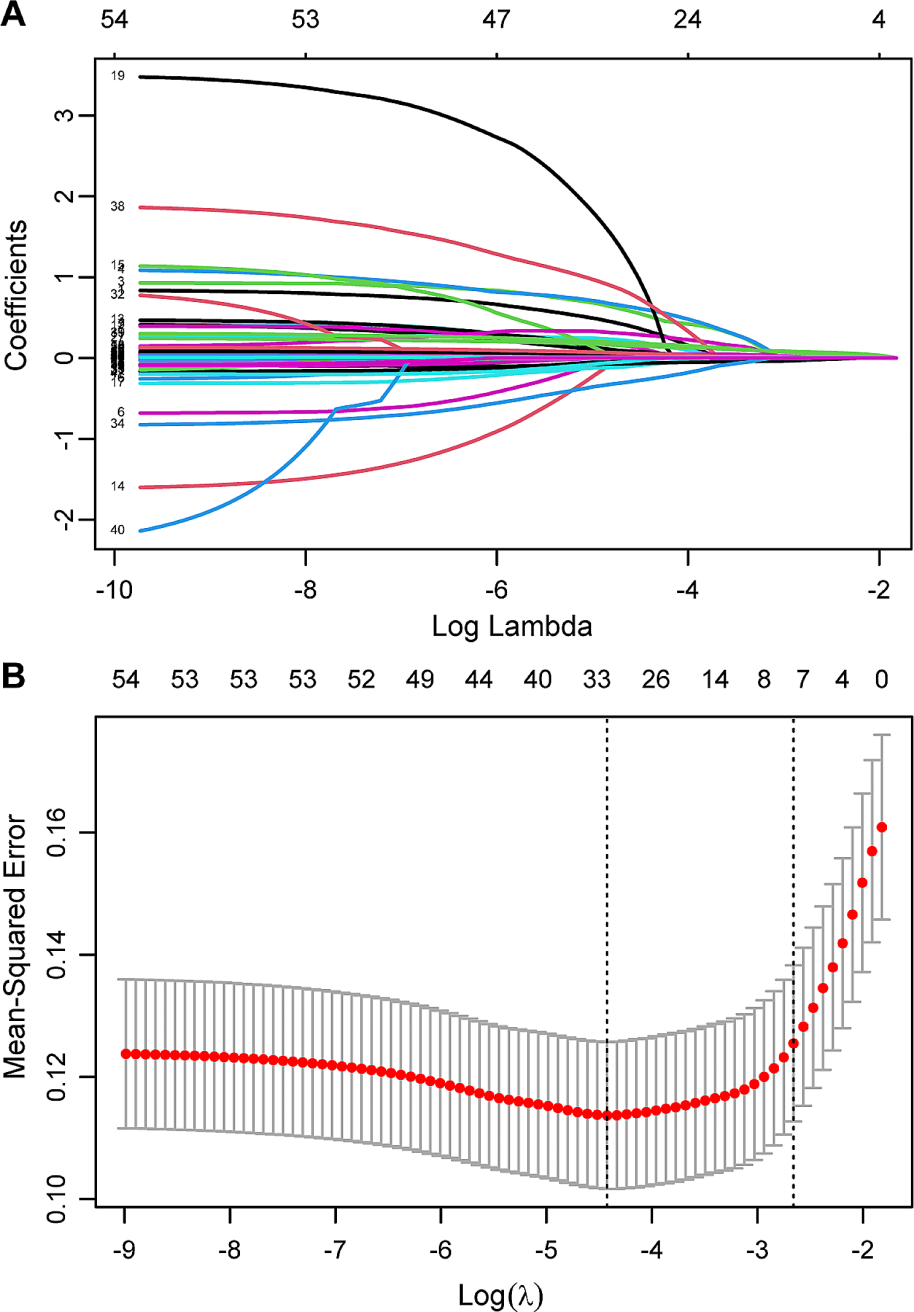
Using Lasso logistic regression model to select clinical variables. (**A**) Tuning parameter (λ) selection using LASSO penalized logistic regression with 10-fold cross-validation. (**B**) Cross validation plot for the penalty term

**Fig. 3:**
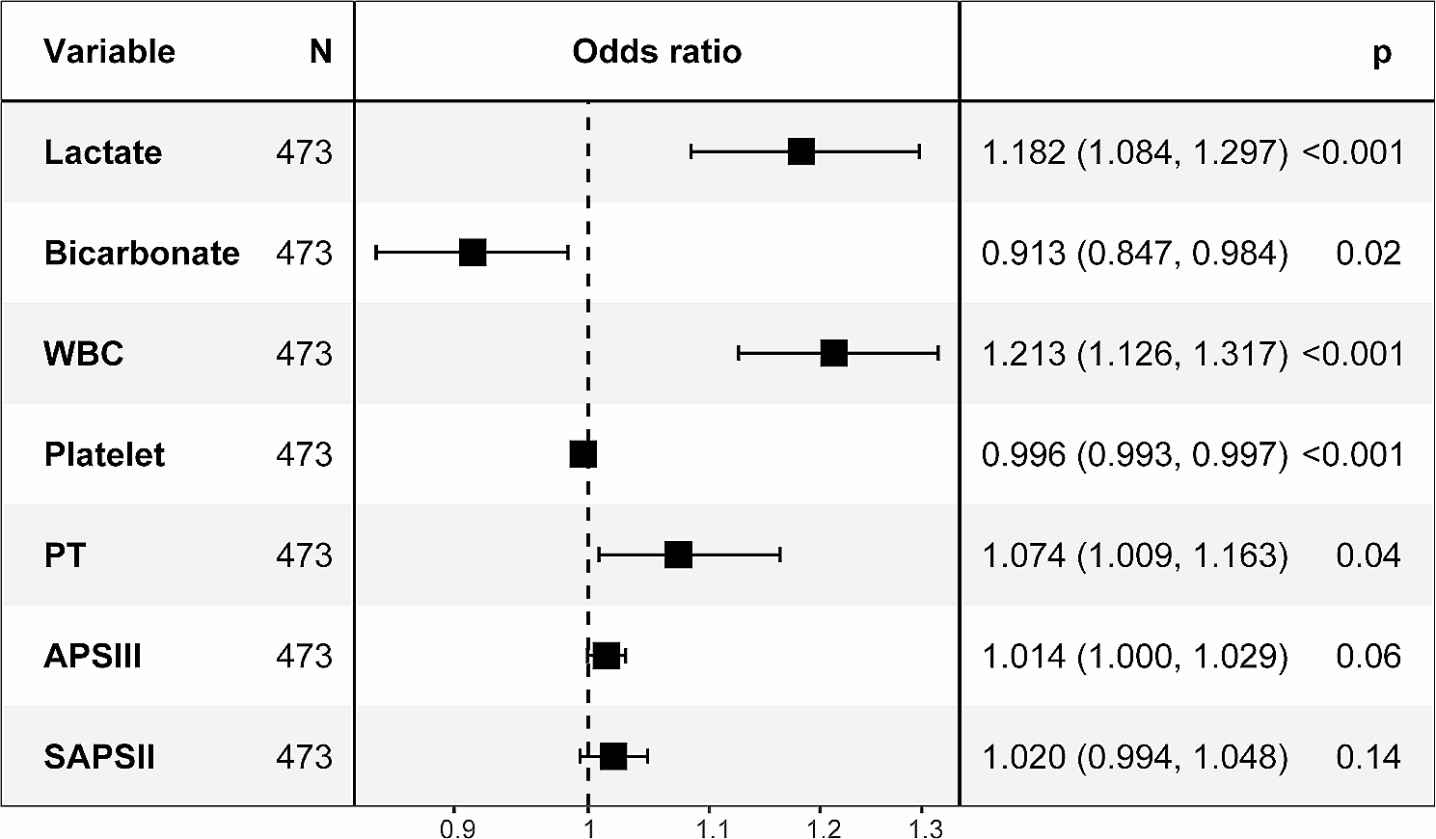
Results of the multiple logistic regression model based on LASSO regression. WBC white blood cell; PT prothrombin time; APSIII acute physiology score III; SAPSII simplified acute physiology score

During the cross-validation process of the LASSO regression, we used the mean square error (MSE) as the evaluation index to successfully screen seven significant independent variables from the initial pool of 54. We selected Lambda.1se and found that the coefficients of these variables were not equal to zero, indicating their significant contribution to the model’s prediction results (Fig. 2). In the multivariate logistic regression analysis, seven variables were incorporated and evaluated: lactate (OR: 1.182; 95% CI 1.084–1.297), bicarbonate (OR: 0.913; 95% CI 0.847–0.984), white blood cells (OR: 1.213; 95% CI 1.126–1.317), platelet (OR: 0.996; 95% CI 0.993–0.997), PT (OR: 1.074; 95%CI 1.009–1.163), APSIII score (OR: 1.014; 95%CI 1–1.029), and SAPSII score (OR: 1.020; 95% CI 0.994–1.048) (Fig. 3).

### Development of the nomogram

**Fig. 4:**
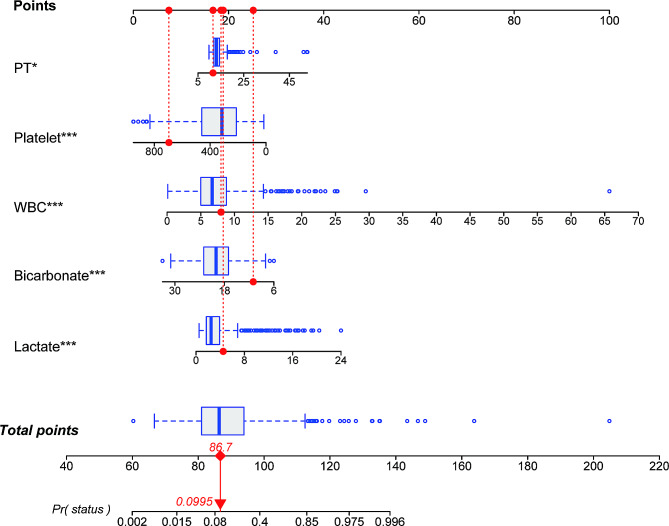
Nomogram for predicting the in-hospital mortality rate of patients with infective endocarditis. Each variable is represented by a vertical line drawn to its corresponding score. The scores for each variable are summed to obtain a total score, which corresponds to the predicted probability of in-hospital mortality rate at the bottom of the nomogram. WBC white blood cell; PT prothrombin time; *** means *p* < 0.001; * means *p* < 0.05

To mitigate the potential impact of multicollinearity on model accuracy, a new nomogram model was constructed by selecting the remaining five variables (Fig. 4), as the APSIII score and SAPSII score overlapped with other variables among the independent variables obtained through LASSO regression. Each factor in the nomogram was assigned an individual score based on its value, and the total score was calculated by summing these scores. The cumulative score derived from the aforementioned parameters was employed in forecasting the mortality of patients with IE during hospitalization in the ICU.

### Evaluation of nomogram performance

**Fig. 5:**
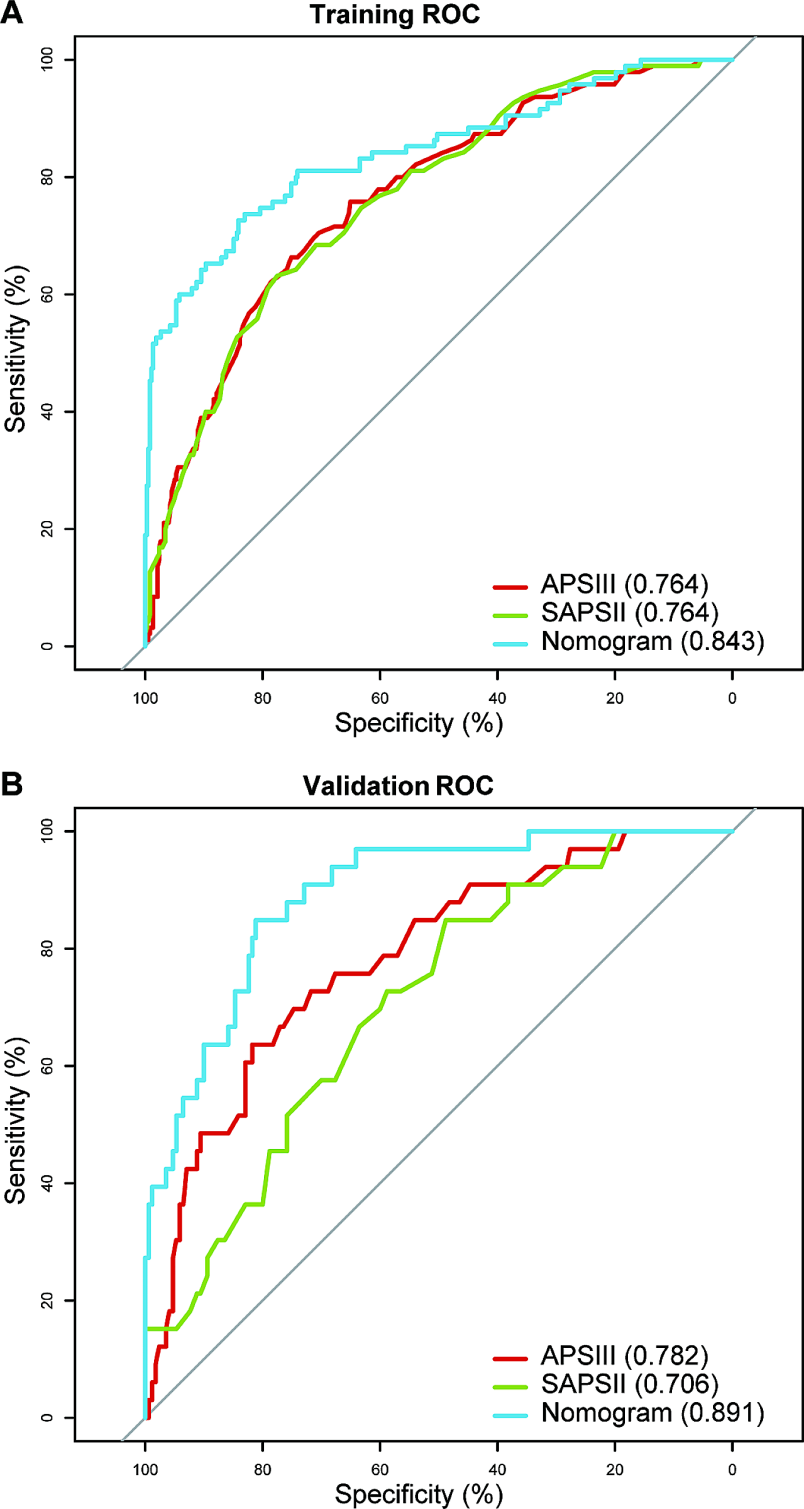
The ROC curves and AUCs of APSIII, SAPSII and nomogram in the training cohort (**A**) and validation cohort (**B**). AUC, area under curve; APSIII acute physiology score III; SAPSII simplified acute physiology score

**Fig. 6:**
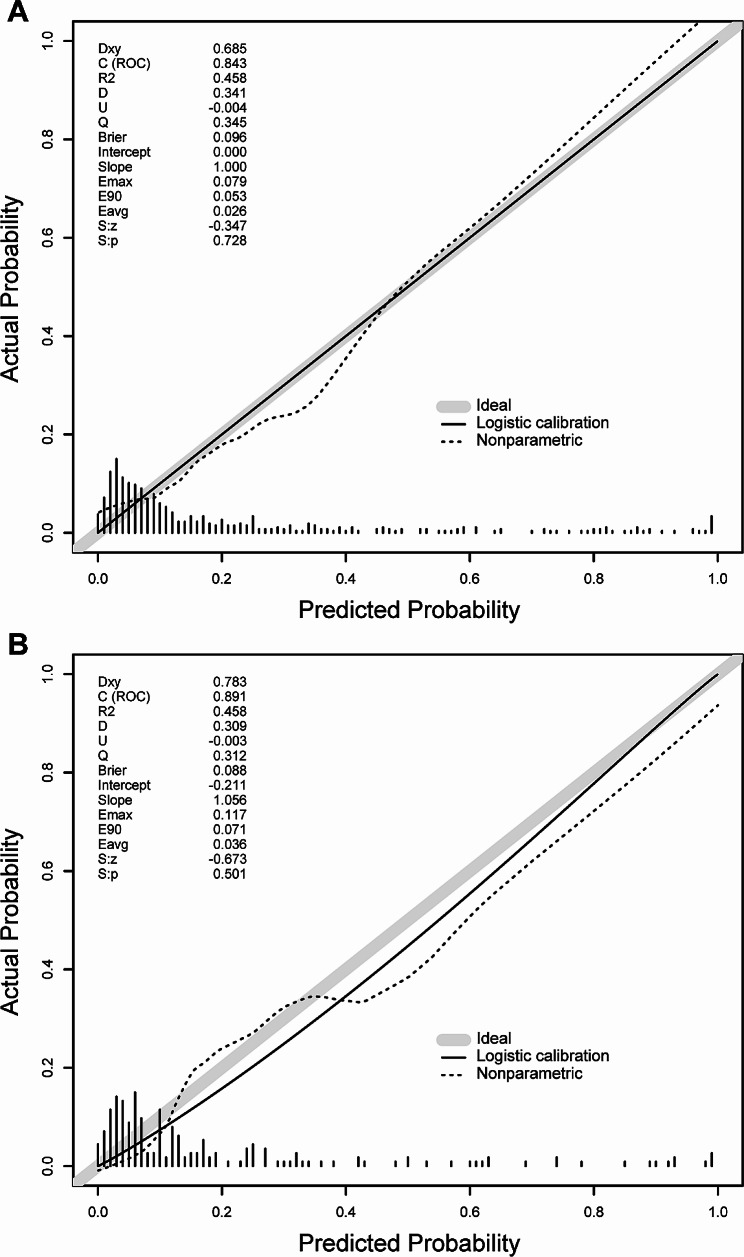
Calibration curves of the training cohort (**A**) and validation cohort (**B**) for the nomogram. The x-axis represents the nomogram-predicted probability, and the y-axis represents the actual probability of the nomogram. The diagonal line represents the perfect prediction of the ideal model. The dashed line represents the nonparametric calibration curve, the solid line represents the calibration curve of logistic regression

**Fig. 7:**
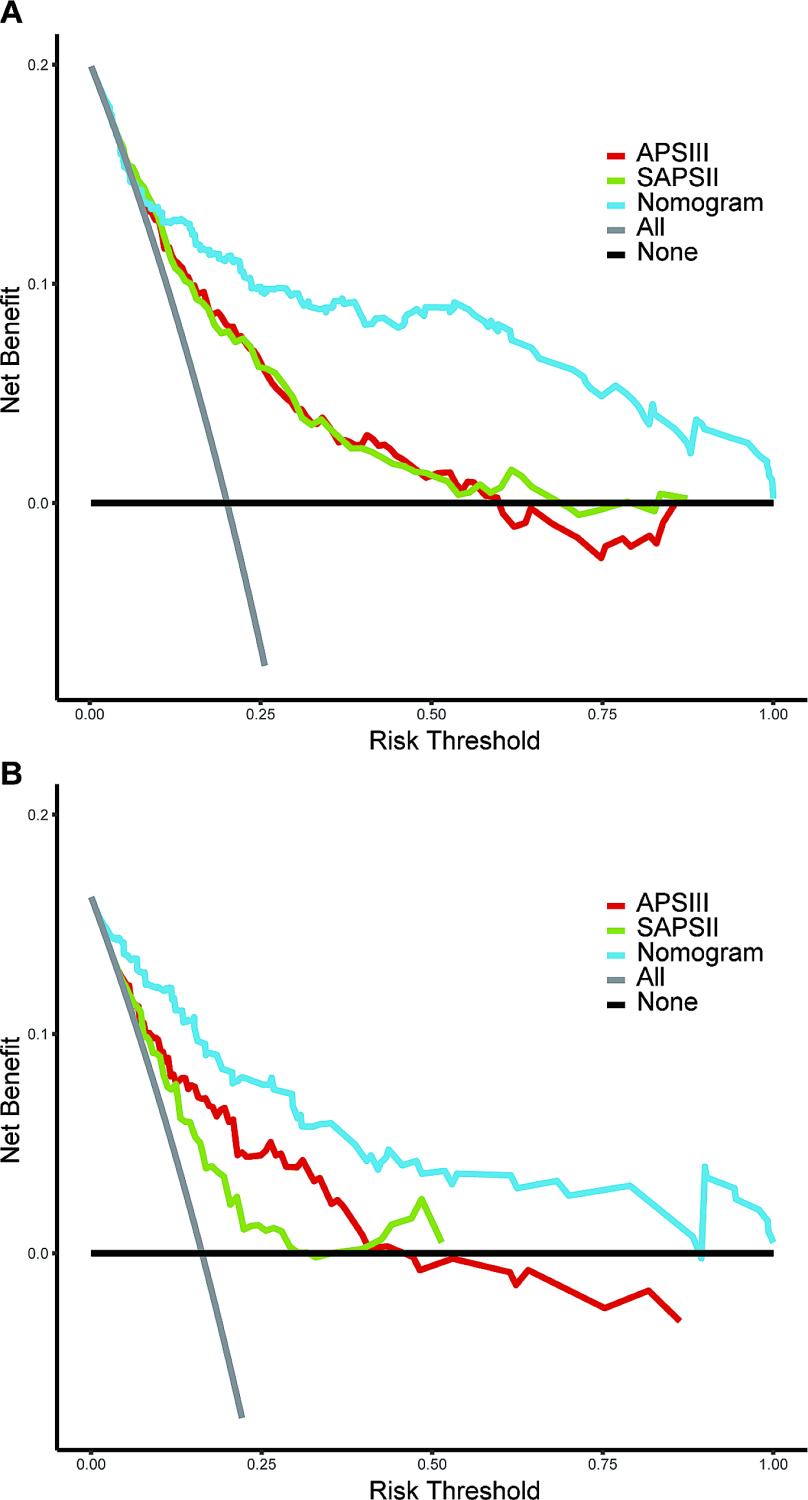
The DCA curve of nomogram, APSIII and SAPSII in the training cohort (**A**) and validation cohort (**B**). The y-axis measures the net benefit. DCA, decision curve analysis; APSIII acute physiology score III; SAPSII simplified acute physiology score

Our nomogram model generated an AUC value of 0.843 (95% CI: 0.792–0.893) under the ROC curve for the training cohort, which is higher than both APSIII (0.764; 95% CI: 0.711–0.817) and SAPSII (0.764; 95% CI: 0.712–0.816). In the validation cohort, the AUC value is also 0.891(95% CI: 0.837–0.946), which is higher than APSIII (0.782; 95% CI: 0.698–0.867) and SAPSII (0.706; 95% CI: 0.617–0.796) (Fig. 5). With a Delong’s test, we have proven that the difference in AUC between our nomogram model and the APSIII and SAPSII scoring systems is statistically significant (Table 2). And calculating the IDI value demonstrates that our model outperforms the two scoring systems, indicating that our nomogram’s graphical model exhibits superior classification accuracy compared to the commonly used APSIII and SAPSII scores (Table 3). In the training and validation sets, the calibration curve closely approximates the ideal curve (45-degree diagonal line). This indicates a strong correlation between the predicted and observed values, signifying a robust fit (Fig. 6). The HL test (subgroups = 10) yielded a χ2 of 13.97 (*p* = 0.12) for the training cohort and 3.68 (*p* = 0.93) for the validation cohort. Finally, the DCA curve indicates that our model has good clinical validity in predicting mortality, as it represents a net benefit (Fig. 7). The DCA curve for the validation cohort indicated a net clinical gain of 8.37% when the prediction probability threshold was set to 20%. These results suggest that our model is more successful in predicting in-hospital mortality caused by IE.

**Table 2:** The AUC of Nomogram, APSIII and SAPSII in training and validation cohort

Predictive Model		AUC	Delong’s test (Z)	*p*-value
Training Cohort	Nomogram	0.843 (0.792, 0.893)		
	APSIII	0.764 (0.711, 0.817)	2.19	0.03
	SAPSII	0.764 (0.712, 0.816)	2.30	0.02
Validation Cohort	Nomogram	0.891 (0.837, 0.946)		
	APSIII	0.782 (0.698, 0.867)	2.51	0.01
	SAPSII	0.706 (0.617, 0.796)	3.60	< 0.001

AUC area under the curve; APSIII Acute Physiology Score III; SAPSII Simplified Acute Physiology Score II. The Delong’s test demonstrated a significant difference in AUC between the APSIII, SAPSII score and the Nomogram model

**Table 3:** The IDI of Nomogram, APSIII and SAPSII in training and validation cohort

Predictive Model		IDI	*p*-value
Training Cohort	Nomogram		
	APSIII	0.241 (0.168, 0.313)	< 0.001
	SAPSII	0.232 (0.161, 0.303)	< 0.001
Validation Cohort	Nomogram		
	APSIII	0.236 (0.106, 0.366)	< 0.001
	SAPSII	0.298 (0.182, 0.413)	< 0.001

IDI integrated discrimination improvement; APSIII Acute Physiology Score III; SAPSII Simplified Acute Physiology Score II. The IDI value demonstrated that Nomogram model exhibited a markedly superior predictive capacity than the APSIII and SAPSII models

## Discussion

The outcome of patients with IE is generally unfavorable, with a high mortality rate [13]. Thus, there is a pressing need for accurate prediction models to identify high-risk patients at an early stage. In this study, we successfully developed a nomogram model for the prediction of in-hospital mortality in IE patients admitted to the ICU, and assessed its effectiveness. Through the utilization of LASSO regression analysis on the data of MIMIC IV database, we identified several significant indicators, namely lactate, bicarbonate, white blood cell count (WBC), platelet count, prothrombin time (PT), APSIII score, and SAPSII score, that were associated with adverse outcomes in this particular patient cohort.

The APS score is a component of the Acute Physiology and Chronic Health Evaluation (APACHE) score. Within APS III, there are 17 physiological variables assessed, including temperature, mean arterial pressure, heart rate, respiratory rate, PaO_2_ or A-aDO_2_, pH or HCO_3_, Na^+^, K^+^, Cr, hematocrit, WBC, blood urea nitrogen, urine output, serum albumin, bilirubin, glucose, and Glasgow Coma Scale (GCS) score [14]. On the other hand, the SAPS II is a tool that incorporates age, admission type (planned surgery, unplanned surgery, or medical), underlying disease variables (acquired immune deficiency syndrome, metastatic cancer, and hematological malignancy), and 12 physiological variables (such as heart rate, systolic blood pressure) into its scoring system [15]. Both APS III and SAPS II scores are commonly used in assessing the risk of mortality in patients [16, 17]. However, due to the overlap of these scoring systems and other variables in this study, the APS III and SAPS II scores were excluded to maintain model accuracy.

Based on these findings, a nomogram prediction model was developed with the aim of providing clinicians with a practical tool to predict in-hospital mortality in patients with IE who are receiving intensive care. By employing this nomogram, physicians can gain a comprehensive understanding of the patient’s condition and identify individuals at high risk during the early stages, enabling the formulation of more personalized treatment plans. Additionally, nomograms can serve as initial assessment tools for patient admission, establish baseline data for patients, and provide references for subsequent treatment and care. To facilitate clinical use, we developed a dynamic nomogram based on this model and made it available on the website, enhancing the user-friendliness of the nomogram prediction model.

Our study found a correlation between lactate elevation and patient mortality. Elevated levels of lactate serve as a crucial prognostic indicator in the evaluation of patients [18], hyperlactatemia strongly associated with poorer prognosis [19]. The mechanisms by which hyperlactatemia occurs have been debated, but irrespective of these mechanisms, numerous studies have demonstrated lactate as a marker for disease severity [20], and there exists a positive correlation between lactate concentration, disease severity and mortality [21, 22]. Bicarbonate serves as a vital regulator of body fluids and acid-base homeostasis, supporting essential physiological processes. However, metabolic acidosis is often observed in patients admitted to Intensive Care Units (ICUs), leading to reduced levels of bicarbonate [23, 24]. Such alteration in bicarbonate levels has been linked to a higher incidence of adverse patient outcomes, including increased morbidity and mortality rates [25].

Coagulation is at the core of IE, with inflammation capable of disrupting the proper balance between the coagulation and immune systems [26]. This disruption leads to the generation of thrombin, which activates platelets and generates fibrin. Fibrin seals infected tissue to prevent further spread of bacteria [27], while platelets with immunoglobulin receptors and pattern receptors can also kill bacteria [28]. Research has revealed that the depletion of platelets can worsen outcomes in animal models of IE [29–31]. Additionally, thrombocytopenia, which is the reduction of platelet counts, is correlated with longer hospital stays, an increased incidence of major bleeding events, and higher in-hospital mortality rate among individuals with septic shock [32]. While platelets could contribute to bacterial adhesion to heart valves that result in vegetation, their involvement in clearing bacteria at a later stage may be more beneficial. The inflammatory response that accompanies IE can cause activated endothelial cells and leukocytes to release several tissue-active factors, which can activate the extrinsic coagulation pathway and prolong PT [26]. Patients with severe infections commonly exhibit elevated white blood cell counts (WBC), and the severity of their illness correlates with the degree of elevation of their WBC counts [33].

The study analyzed several known risk factors that could affect the prognosis of IE patients, such as CRRT, blood culture results, embolism symptoms and prior history of cardiac surgery [34], but none of these variables were incorporated into the model. This situation can be attributed to the requirement of producing a practical and reliable model. Therefore, Lambda.1se was utilized as the cut-off point for variable selection during LASSO regression to avoid overfitting the model with too many selected variables.

In clinical practice, several scoring systems, including ANCLA, PALSUSE, DeFeo, RISK-E and EndoSCORE have been employed to evaluate the prognosis of patients with IE [35–39], these scoring systems mainly focus on the postoperative prognosis of IE surgery patients. This approach is inadequate, as only approximately 51% of IE patients undergo surgical intervention, as shown in EURO-ENDO 2019 registry data [40]. Our study exhibits a significant advantage due to the comprehensive inclusion of data pertaining to all patients diagnosed with IE who were admitted to the ICU, as opposed to solely concentrating on those necessitating surgical intervention. In addition, the variables present in our model comprise of objective indicators widely utilized in routine clinical practice, hence facilitating the process of data acquisition.

Our study also has several limitations. First, echocardiographic findings could not be included in the analysis as the MIMIC IV 2.0 database did not have such imaging data. Second, the data was gathered solely from a single medical center, which may hinder the generalizability of the findings to a broader population. Moreover, we only performed internal validation of the model, indicating the need for further research involving external validation to consider other factors and improve the model’s validation.

## Conclusions

We developed a practical nomogram model based on laboratory results, primarily comprising lactate, bicarbonate, WBC, platelet, PT. Our model demonstrates a precise estimation of in-hospital mortality among patients with IE within the ICU setting. The objective of the model is to assist physicians in making reasonable assessments and treatments, resulting in an improved survival rate of patients while hospitalized.

### Electronic supplementary material

The webpage prediction tool corresponding to the column chart we are studying is located at (https://cdyhaa.shinyapps.io/IENomapp/).

Below is the link to the electronic supplementary material.

Supplementary Material 1

## Data Availability

The data were available on the MIMIC-IV website at https://mimic.physionet.org/, 10.13026/a3wn-hq05.

## Abbreviations

AF: Atrial fibrillation
AG: Anion gap
ALT: Alanine transaminase
APSIII: Acute physiology score III
AUC: Area under the curve
CRRT: Continuous renal replacement therapy
DBP: Diastolic blood pressure
HR: Heart rate
IDI: Integrated Discrimination Improvement
LASSO: Least Absolute Shrinkage and Selection Operator
LD: Lactic dehydrogenase
MBP: Mean blood pressure
MCH: Mean corpuscular hemoglobin
MIMIC IV: Medical Information Mart for Intensive Care IV
PT: Prothrombin time
PTT: Partial thromboplastin time
RBC: Red blood cell
SAPSII: Simplified acute physiology score
SBP: Systolic blood pressure
SOFA: Sequential organ failure assessment
WBC: White blood cell
