# Supplementary material for: Development and validation of a nomogram for predicting in-hospital mortality in ICU patients with infective endocarditis
Source: BMC Med Inform Decis Mak. 2024 Mar 21;24:84. doi: 10.1186/s12911-024-02482-7 (PMC10958908; doi:10.1186/s12911-024-02482-7)
Supplement: Supplementary file 1 — Supplementary Material 1 [file 12911_2024_2482_MOESM1_ESM.docx]

**Table 1** Baseline characteristics of the patients（Supplementary）

| **Variables** | **Training Cohort** | **Validation Cohort** | ***p*-value** |
| --- | --- | --- | --- |
| **Complication** |  |  |  |
| Myocardial infarct |  |  | 0.836 |
| No | 389 (82.2%) | 169 (83.3%) |  |
| Yes | 84 (17.8%) | 34 (16.7%) |  |
| Congestive heart failure |  |  | 0.712 |
| No | 245 (51.8%) | 109 (53.7%) |  |
| Yes | 228 (48.2%) | 94 (46.3%) |  |
| Cerebrovascular disease |  |  | 0.656 |
| No | 321 (67.9%) | 142 (70.0%) |  |
| Yes | 152 (32.1%) | 61 (30.0%) |  |
| Chronic pulmonary disease |  |  | 0.613 |
| No | 353 (74.6%) | 147 (72.4%) |  |
| Yes | 120 (25.4%) | 56 (27.6%) |  |
| Liver disease |  |  | 0.044 |
| No | 359 (75.9%) | 169 (83.3%) |  |
| Yes | 114 (24.1%) | 34 (16.7%) |  |
| Diabetes |  |  | 1.000 |
| No | 338 (71.5%) | 145 (71.4%) |  |
| Yes | 135 (28.5%) | 58 (28.6%) |  |
| Renal disease |  |  | 0.613 |
| No | 353 (74.6%) | 147 (72.4%) |  |
| Yes | 120 (25.4%) | 56 (27.6%) |  |
| Malignant cancer |  |  | 0.128 |
| No | 438 (92.6%) | 180 (88.7%) |  |
| Yes | 35 (7.40%) | 23 (11.3%) |  |
| Metastatic solid tumor |  |  | 0.069 |
| No | 465 (98.3%) | 194 (95.6%) |  |
| Yes | 8 (1.69%) | 9 (4.43%) |  |
| AIDS |  |  | 0.291 |
| No | 465 (98.3%) | 202 (99.5%) |  |
| Yes | 8 (1.69%) | 1 (0.49%) |  |
| AF |  |  | 0.368 |
| No | 344 (72.7%) | 140 (69.0%) |  |
| Yes | 129 (27.3%) | 63 (31.0%) |  |
| Valve disease |  |  | 0.619 |
| No | 330 (69.8%) | 137 (67.5%) |  |
| Yes | 143 (30.2%) | 66 (32.5%) |  |
| **Risk factors** |  |  |  |
| CRRT |  |  | 1.000 |
| No | 438 (92.6%) | 188 (92.6%) |  |
| Yes | 35 (7.40%) | 15 (7.39%) |  |
| Embolism |  |  | 0.650 |
| No | 390 (82.5%) | 171 (84.2%) |  |
| Yes | 83 (17.5%) | 32 (15.8%) |  |
| Postcardiac surgery |  |  | 0.215 |
| No | 387 (81.8%) | 157 (77.3%) |  |
| Yes | 86 (18.2%) | 46 (22.7%) |  |
| Blood culture |  |  | 0.777 |
| Negative | 303 (64.1%) | 127 (62.6%) |  |
| Positive | 170 (35.9%) | 76 (37.4%) |  |
| Congenital heart disease |  |  | 0.681 |
| No | 467 (98.7%) | 202 (99.5%) |  |
| Yes | 6 (1.27%) | 1 (0.49%) |  |

AF atrial fibrillation; CRRT continuous renal replacement therapy
